# Cabotegravir, the Long-Acting Integrase Strand Transfer Inhibitor, Potently Inhibits Human T-Cell Lymphotropic Virus Type 1 Transmission *in vitro*

**DOI:** 10.3389/fmed.2022.889621

**Published:** 2022-04-25

**Authors:** Bethany S. Schneiderman, Michal S. Barski, Goedele N. Maertens

**Affiliations:** Department of Infectious Disease, Imperial College London, London, United Kingdom

**Keywords:** HTLV-1, MTCT, integrase, INSTI, cabotegravir, tenofovir disoproxil fumarate, pre-exposure prophylaxis

## Abstract

Human T-cell lymphotropic virus type 1 (HTLV-1) is a deltaretrovirus most prevalent in southwestern Japan, sub-Saharan Africa, Australia, South America, and the Caribbean. Latest figures approximate 10 million people worldwide to be infected with HTLV-1. This is likely a significant underestimation due to lack of screening in endemic areas and absence of seroconversion symptoms. The two primary diseases associated with HTLV-1 infection are adult T cell leukaemia-lymphoma, a malignant and, sometimes, aggressive cancer; and HTLV-1 associated myelopathy/tropical spastic paraparesis, a debilitating neurological degenerative disease. Unfortunately, despite the poor prognosis, there is currently no effective treatment for HTLV-1 infection. We previously showed that integrase strand transfer inhibitors (INSTIs) clinically used for human immunodeficiency virus type 1 (HIV-1) prophylaxis and treatment are also effective against HTLV-1 transmission *in vitro*. In 2021 a new INSTI, cabotegravir, was approved by the FDA for HIV-1 treatment. We thus set out to evaluate its efficacy against HTLV-1 infection *in vitro*. Strand transfer assays performed using recombinant HTLV-1 integrase treated with increasing concentrations of cabotegravir, effectively inhibited strand transfer activity, displaying an IC_50_ of 77.8 ± 22.4 nM. Furthermore, cabotegravir blocked HTLV-1 transmission in tissue culture; we determined an EC_50_ of 0.56 ± 0.26 nM, similar to bictegravir. Alu-PCR confirmed the block in integration. Thus, there are four INSTIs and one reverse transcriptase inhibitor approved by the FDA for HIV-1 treatment, that potently block HTLV-1 infection *in vitro*. This should strongly encourage the establishment of a new standard of HTLV-1 treatment – particularly for pre-exposure prophylaxis and prevention of mother-to-child transmission.

## Introduction

Three decades ago, infection with human immunodeficiency virus type 1 (HIV-1), a close relative of human T-cell lymphotropic virus type 1 (HTLV-1), was almost universally fatal. Introduction of antiretroviral therapy with drugs targeting the viral reverse transcriptase (RT) enzyme and protease enzymes, seropositive patients got a second chance to live. Since 2007, a novel class of inhibitors, targeting the viral integrase (IN) enzyme, integrase strand transfer inhibitors (INSTIs) completed the current list of approved antiretrovirals (1, 2). HIV-1 RT is error-prone; this, in addition to the high rate of viral replication and high viral load, gives rise to a large number of quasi-species that can be circulating within one host at any time (3). Generation of drug resistant HIV-1 escape mutants is high, given the propensity of RT to mutate and further promoted by low adherence to a strict drug regimen. Consequently, due to monotherapy treatment proving unsuccessful, combined anti-retroviral therapy (cART) has been the standard treatment option for HIV-1 carriers. Careful monitoring of the viral load continues to be important to detect whether (multi-)drug resistant viruses develop over the course of time.

Human T-cell lymphotropic virus type 1, discovered shortly before HIV-1 (4), infects around 10 million people worldwide (5). At least for the majority of HTLV-1 carriers, infection is not universally fatal. Indeed, ∼5% of HTLV-1 infected individuals develop an aggressive form of leukaemia, adult T-cell lymphoma/leukaemia (ATLL), and, when acute, will die within 8 months from presentation (6). HTLV-1 associated myelopathy/tropical spastic paraparesis affects another ∼5% of HTLV-1 carriers and results in a debilitating neurodegenerative disease where most affected patients become wheelchair bound, having to live in constant pain and have their social and economic status severely degraded. In addition to ATLL and HAM/TSP, HTLV-1 is known to cause a wide range of other inflammatory diseases (7), is the main risk factor for the development of severe strongyloidiasis (8), and HTLV-1 carriers have a substantially elevated risk of developing a further 16 diseases (9). Most strikingly, a recent study showed that most otherwise asymptomatic carriers suffer from ailments that severely impact their quality of life, including pain, general discomfort, and depression, indicating a much greater impact on society (including health care costs) than previously anticipated (10). Current treatments for HTLV-1 associated diseases are limited and often poorly effective; improved therapies are urgently required.

Developing disease is positively correlated with the proviral load (PVL) (≥4%), and in the case for ATLL, an oligoclonality index determined by flow cytometry >0.77 increases the risk of developing disease (11). Use of condoms are advised for people with HTLV-1 to reduce the likelihood of sexual transmission between serodiscordant couples. Mother-to-child transmission (MTCT) which contributes to about 24% of HTLV-1 transmission events, can be partially prevented by, e.g., reducing the time of breastfeeding (<6 months), freeze-thawing the breastmilk and/or using formula (12–15). However, the pressure on women to breastfeed their children, either due to cultural or environmental factors, or due to lack of access to a freezer or clean water, often prevents this type of intervention.

We previously reported on the efficacy of INSTIs in blocking HTLV-1 transmission *in vitro* (16, 17). Most recently, we also identified a novel naphthyridine compound XZ450 which efficiently blocks HTLV-1 integration in cells (17). The development of the long-acting drug cabotegravir (CAB), which allows for a regimen of (bi-)monthly injections has the potential to greatly affect the quality of life of people living with HIV-1, such as serodiscordant couples where both the seronegative partner could use CAB as pre-exposure prophylaxis (PrEP) and/or the seropositive partner could use CAB-based treatment as prevention, as well as patients that are less likely to adhere to a strict and regular drug regimen.

We thus thought to investigate how efficiently CAB can block HTLV-1 infection and integration. Here, we report our findings and show that CAB is a highly potent inhibitor of HTLV-1 infectivity in tissue culture, with an EC_50_ similar to bictegravir (BIC) (16) in the low nanomolar range.

## Methods

### Measurement of Human T-Cell Lymphotropic Virus Type 1 *in vitro* Strand-Transfer Activity

**FIGURE 1:**
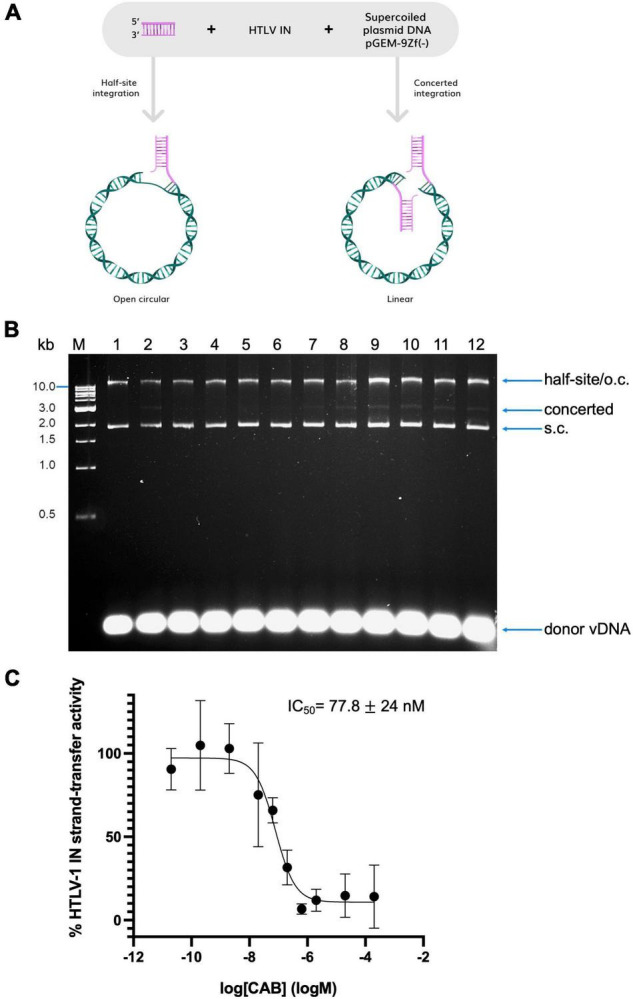
Inhibition of in vitro strand transfer activity of recombinant HTLV-1 IN by CAB. **(A)** Schematic illustrating the strand-transfer assay. Products of half-site integration co-migrate with open circular forms of DNA; concerted integration linearizes the supercoiled target DNA. **(B)** Representative gel shown used to quantify IN inhibition by CAB as shown in panel **(C)**. Precipitated DNA samples were analysed on an agarose gel stained with ethidium bromide. Lane 1, negative control: no IN; lane 2: positive control, IN in the absence of drug. Lanes 3–12: in the presence of CAB; lane 3: 200 μM, lane 4: 20 μM, lane 5: 2 μM, lane 6: 634 nM, lane 7: 200 nM, lane 8: 63.4 nM, lane 9: 20 nM, lane 10: 2 nM, lane 11: 200 pM, and lane 12: 20 pM. Migration of DNA species in the gel is indicated on the right of the gel. S.c., supercoiled; o.c., open circular. The 1 kb DNA ladder (NEB, indicated on the left of the gel) was used as a reference. **(C)** Dose-response curve of CAB calculated based on the amount of concerted integration activity of HTLV-1 IN quantified by densitometry. Averages and standard deviations of three independent replicates are shown.

Human T-cell lymphotropic virus type 1 integrase (IN) was purified as reported previously (17). Inhibition of HTLV-1 IN *in vitro* strand-transfer activity was measured by means of a strand-transfer assay. A schematic illustrating the assay is shown in Figure 1A. Oligonucleotides mimicking the U5 3′-processed LTR ends of the viral DNA (vDNA) copy (H1U5_S20UP and H1U5_S20B, Supplementary Table 1), were firstly annealed in 100 mM Tris pH 7.4, 400 mM NaCl. The reaction mixture contained 73 mM PIPES pH 6.0, 175 mM NaCl, 16.7 mM MgCl_2_, 5.8 μM ZnCl_2_, 12.8 mM DTT, 0.53 μM donor vDNA, 1.6 μM HTLV-1 IN. This was then incubated in the presence of a given concentration of CAB (dissolved in DMSO) or with DMSO alone (final concentration in the reaction mixture was 5%) for 30 min at room temperature. A total of 300 ng of target DNA [supercoiled pGEM-9Zf(-)] was then added to initialise the reaction, which was carried out at 37°C for 30 min. Once completed, samples were deproteinised and DNA products precipitated as described previously (16, 18). Resultant DNA sample was loaded on a 1.5% agarose gel and analysed after staining with ethidium bromide. Experiments were conducted in triplicate. Bands corresponding to products of concerted integration were quantified by densitometry in ImageLab 4.1 (Bio-Rad). Dose-response curve fitting was done in Prism 8. Cumulative standard deviation for each drug was calculated as an average of the upper limit and lower limit values:


$$\mathrm{Lower}\,\mathrm{limit}=\frac{I\,C_{50}}{10^{\log S\,E\,\left(I\,C_{50}\right)}};U\,p\,p\,e\,r\,l\,i\,m\,i\,t=I\,C_{50}\times 10^{\log S\,E\,\left(I\,C_{50}\right)}$$

$$\text{SD}=\frac{\left[\left(U\,p\,p\,e\,r\,l\,i\,m\,i\,t-I\,C\,50\right)+\left(I\,C\,50-l\,o\,w\,e\,r\,l\,i\,m\,i\,t\right)\right]}{2}$$

where SE denotes the standard error computed by Prism.

### Human T-Cell Lymphotropic Virus Type 1 Infection

The MT-2 and Jurkat (E6.1) T cell lines (ATCC) were maintained in Roswell Park Memorial Institute (RPMI) media supplemented with 10% heat inactivated foetal bovine serum (Gibco), 100 U/mL penicillin, 100 μg/mL streptomycin, and 0.25 μg/mL fungizone (Gibco) (referred to as complete medium). Cells were cultured in a humidified atmosphere at 37°C with 5% CO_2_. HTLV-1 infection was conducted as previously outlined (16, 17). In brief, Jurkat E6.1 cells were seeded in complete medium and pre-treated with CAB ranging from 200 nM to 8 fM, or DMSO (CAB vehicle), for 24 h. MT-2 cells were exposed to a sub-lethal dose of gamma-irradiation (400 Gray) and resuspended in serum free media to 2e^6^ cells/mL. Pre-treated Jurkat cells were resuspended in serum free media to 2e^6^ cells/mL. A total of 250 μL of both the producer cell line (MT-2) and the target cell line (Jurkat cells) were co-cultured in serum free medium, in the presence of increasing concentrations of CAB and 15 mM MgCl_2_. This resulted in a culture of 0.5e^6^ of each cell type in a total of 500 μL. Following a 17.5-h infection, cells were washed with PBS and mixed with 25 μL magnetic anti-CD25 beads (DynaBeads, Thermo Fisher Scientific) to enable depletion of MT-2 cells from the culture (16, 17). Both the beads and cells were equilibrated in 1 mL depletion buffer (1× PBS + 0.1% FBS + 2 mM EDTA). Cells were gently tumbled for 1 h at 4°C. The resultant mixture was applied twice to a magnet and unbound supernatant was resuspended in 500 μL complete media in the presence of CAB or DMSO. Cells were maintained for 12 days in the presence of drug and DNA was harvested on day 13 (DNeasy Kit, Qiagen). Data of four independent biological replicates are shown. Depletion of MT-2 cells was optimised and verified by FACS as described previously (16). Cell viability was monitored throughout the duration of the experiment by trypan blue exclusion.

### Quantification of Human T-Cell Lymphotropic Virus Type 1 Proviral Load and Integrated DNA

Proviral load was determined as described previously (16, 19, 20), with the modification that we used Taqman PCR rather than SYBR green incorporation. To this end, extracted gDNA was quantified using a spectrophotometer (DeNovix) and gDNA was diluted to 5 ng/μL. A total of 20 ng of gDNA was loaded in each TaqMan qPCR reaction, using the primers for HTLV-1 *tax* gene and human *ALBUMIN*. Copy numbers of each gene were quantified *via* comparison to standard curves generated from clone 11.50 (a kind gift from Professor Charles Bangham; 11.50 is a clone with a known single integration site), or uninfected control, respectively. With the knowledge that there is one copy of *tax* gene and two copies of *ALBUMIN* per cell, PVL was calculated [(2 × tax)/albumin]. Data was normalised to DMSO, which was set to 100%. Dose-response curve fitting was done in Prism 8. Cumulative standard deviations were calculated as described above for the *in vitro* IC_50_ data. The 200 nM and DMSO samples underwent Alu-qPCR to quantify integrated DNA, as previously explained (16), with the alteration of using TaqMan reagents (Supplementary Table 1). Integrated provirus copy numbers were normalised to *ALBUMIN*. Data were normalised to DMSO, which was set to 100%. Averages and standard deviations of four independent biological replicates are shown. *p*-Values were calculated using the Student’s *t*-test.

## Results and Discussion

We first tested the ability of CAB to inhibit HTLV-1 IN strand transfer activity *in vitro*. Thus, strand transfer assays (Figure 1A) were performed using recombinantly purified HTLV-1 IN in the presence of a range of CAB concentrations (micromolar to picomolar range), or its vehicle DMSO. DNA was separated by agarose gel electrophoresis (Figure 1B). Upon concerted integration of the two vDNA mimics, the supercoiled plasmid target DNA migrates as a linear form and can be readily separated from the target DNA as indicated on the figure. Products of concerted strand transfer activity were quantified by densitometry as described previously (16). CAB potently inhibits HTLV-1 IN strand transfer activity and has an IC_50_ of 77.8 ± 24.2 nM (Figure 1C), similar to elvitegravir and BIC (16) (Table 1).

**TABLE 1:** Calculated parameters of *in vitro* HTLV-1 IN strand-transfer and HTLV-1 infection inhibition by INSTIs and one NRTI.

Antiretroviral	IC_50_ ± SD^a^ (nM)	EC_50_ ± SD^b^ (nM)
Raltegravir	530 ± 102^c^	6.42 ± 4.24^c^
Elvitegravir	100 ± 16^c^	9.57 ± 5.54^c^
Bictegravir	180 ± 29^c^	0.302 ± 0.173^c^
Cabotegravir	78 ± 24	0.557 ± 0.255
Tenofovir disoproxil fumarate	NR	17.78 ± 7.16^c^

*^a^IC_50_ = half maximal inhibitory concentration. ^b^EC_50_ = half maximal effective concentration. ^c^Barski et al. (16).*

**FIGURE 2:**
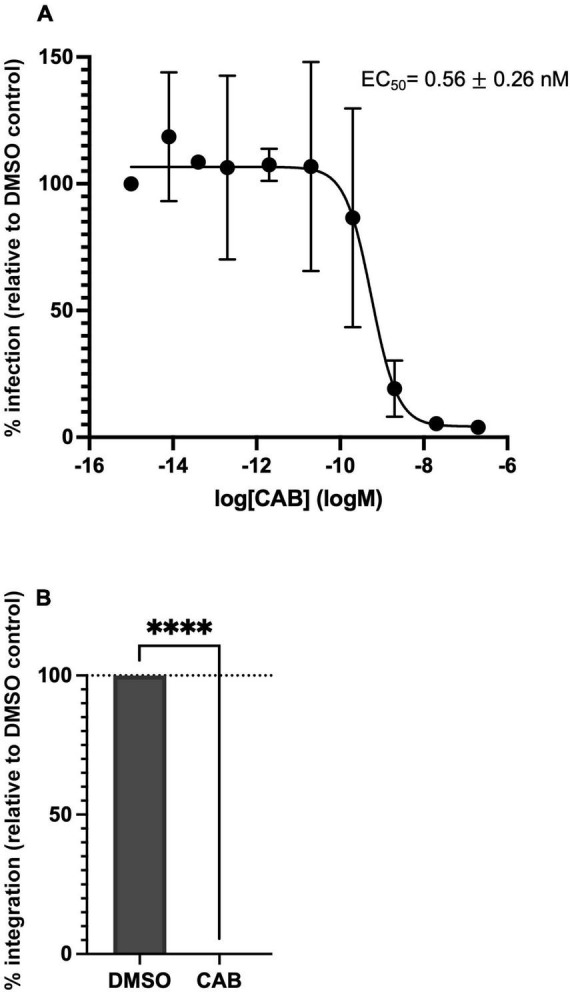
Cabotegravir potently blocks HTLV-1 infection and integration in tissue culture. **(A)** Infection was quantified by measuring the PVL of INSTI treated cells compared to DMSO treated cells arbitrarily set at 100%. Averages and standard deviations of four independent experiments are shown. **(B)** Integrated provirus of DMSO and 200 nM CAB (CAB) treated samples was quantified by Alu-qPCR and normalised to *ALBUMIN* numbers. Values are relative to DMSO control. Average and standard deviations of four independent experiments are shown; ^****^p-value < 0.0001.

To investigate its ability to suppress HTLV-1 transmission in tissue culture, we performed HTLV-1 infection of Jurkat CD4^+^ T cells by co-culture with the persistently HTLV-1 infected MT-2 cell line (16, 17). To this end, gamma-irradiated MT-2 cells were co-cultured with Jurkat cells in the presence of DMSO (vehicle) or a range of CAB concentrations (8 fM–200 nM). Productive infection of Jurkat cells was measured by determining PVL (16, 17, 21). CAB very effectively blocked HTLV-1 transmission; we determined an EC_50_ of 0.56 ± 0.26 nM (Figure 2A and Table 1). A block in integration was confirmed by measuring the products of integration using Alu-qPCR (16) (Figure 2B). CAB thus inhibits HTLV-1 infection *in vitro* as effectively as BIC (Table 1). Similar inhibition profiles for CAB and BIC were also reported for HIV-1 (22).

It is important to note that the much higher observed IC_50_ values in *in vitro* reactions compared to our observed EC_50_ values in infection assays is expected. Indeed, the concentration of IN in the strand transfer assays is much greater than in the context of an infected cell. Thus, the IC_50_/EC_50_ ratio of CAB is ∼139, which is similar to raltegravir (∼83). BIC, however, is much more potent in cell culture (16), with an IC_50_/EC_50_ ratio of 600. Whether this is due to more efficient cellular uptake of BIC or because this compound influences HTLV-1 transmission in an additional fashion requires further investigation.

Cabotegravir is the latest addition to the list of potent INSTIs capable of blocking HIV-1 transmission and replication. Unlike the other INSTIs, the half-life of intramuscularly administered CAB is exceptionally long, typically ranging from 40 to 55 days (23, 24). Reportedly, a single dose of 800 mg intramuscularly delivered CAB achieved a mean concentration above the protein adjusted IC_90_ of approximately 16 weeks (23). This makes CAB attractive for monthly or even less frequent injections for patients who, e.g., poorly adhere to other cART regimens and are therefore more likely to develop drug-resistant HIV, as well as for use as PrEP. For the treatment of HIV-1 infection, CAB is usually offered in combination with rilpivirine (RPV), a non-nucleoside reverse transcriptase inhibitor that is inactive against HTLV-1. Given that HTLV-1 is much less prone to genetic drift than HIV-1 (25), and therefore less likely to develop resistance, the use of CAB for monotherapy especially as a PrEP, including to prevent sexual transmission of HTLV-1, seems appealing.

Whilst there are multiple factors that influence MTCT, and even more questions that require urgent answers [excellently reviewed by Rosadas and Taylor (26)] we do know that exposure to HTLV-1 early on in life has been associated with an increased risk of developing ATLL (27–29), HAM/TSP, and infective dermatitis associated with HTLV-1 (IDH) (30–33). In fact, IDH in children is associated with high PVL and an increased risk of developing HAM/TSP and ATL, often with an onset during young adulthood.

At present, there is limited knowledge on the pharmacokinetics of CAB in pregnant women. Indeed, data is available from 26 women receiving CAB + RPV who became pregnant during clinical trials (34). There were six miscarriages [of which five in the first 9 weeks of gestation which is close to the ∼17–22% reported early pregnancy losses in HIV-1 uninfected women (35)], 9 elective terminations, and of the 11 live births, 1 was reported with a congenital anomaly. Although obviously more research is necessary, it is encouraging that the women becoming pregnant on CAB had adequate CAB concentrations throughout pregnancy as well as post-delivery. Nevertheless, careful monitoring of birth defects following antiretroviral therapy during pregnancy is critical, as well as more research is required to determine the pharmacokinetics of CAB on neonates and maternal breast milk.

A reduction of the PVL in baboons naturally infected with simian T-cell lymphotropic virus type 1 (STLV-1) was achieved through combination therapy with zidovudine (RT-inhibitor) and the HDAC inhibitor valproic acid (36). In humans, mostly zidovudine in combination with IFN-alpha or valproic acid has been used to treat HTLV-1 patients with some forms of ATLL, and was shown to reduce PVL in some cases (37–39). A recent report however, suggests ATLL patients treated with zidovudine/IFN-alpha are more likely to present with invasive fungal infections, which for patients with the aggressive subtype of ATLL resulted in lower overall survival (40). The exact mechanism of how zidovudine/IFN-alpha/valproic acid reduces the PVL in ATLL patients is unknown; data suggests that it is more likely due to induction of cellular senescence and apoptosis of HTLV-1 infected cells rather than inhibition of RT (41, 42). Thus, whilst the use of zidovudine has shown promise in a few cases of ATLL, more studies are necessary to establish whether antiretrovirals are efficacious in reducing PVL in HTLV-1 carriers and can impact the development of IDH, HAM/TSP and/or ATLL.

Human T-cell lymphotropic virus type 1 infection dynamics is different from HIV-1. HIV-1 continues to actively replicate in infected carriers. By contrast, following initial weeks of active viral replication, the HTLV-1 proviral burden is maintained mainly through clonal expansion of the infected lymphocytes. Although viral proliferation continues, this happens at a much slower rate than the observed mitotic spread (43). Thus, the most promising use of antiretrovirals could be pre- or immediate (h/days) post-exposure intervention. We know indeed that the time frame to administer antiretrovirals to recently exposed HTLV-1 patients is very narrow (44). Zidovudine can block HTLV-1 infection somewhat (<10–20% inhibition at 50 μM concentration) (45). We reported previously that tenofovir disoproxil fumarate (TDF), a nucleoside RT-inhibitor (NRTI), efficiently blocks HTLV-1 transmission in cell culture (EC_50_ = 17.8 ± 7.2 nM) (16). Therefore, there is scope to possibly prevent HTLV-1 transmission in the first place by using TDF and INSTIs as PrEP.

Japan is the only country where pregnant women are screened for HTLV-1, and infected mothers are closely monitored and strongly advised to formula feed their babies (14). Although not 100% effective, this approach has shown significant reduction in MTCT [down to 2.4–3.6% in formula fed children compared to up to 24% in breastfed children (12, 13, 15)]. This suggests that (a) perinatal transmission might contribute in part to MTCT, it was shown indeed that HTLV-1 infects placental trophoblasts (46); and (b) MTCT can be blocked and that will have a huge impact on society given that up to 24% of children seroconvert upon prolonged (>6 months) breastfeeding.

From HIV-1 research, we know that both the pregnant woman and the developing embryo are safe when the mother uses RAL or TDF during pregnancy (47, 48). We have shown high efficacy of inhibiting HTLV-1 transmission by four FDA approved INSTIs (raltegravir, elvitegravir, BIC, and CAB) as well as one FDA approved NRTI, TDF (Table 1). Whilst more research is required to understand the pharmacokinetics, safety, and efficacy of blocking MTCT by using BIC and CAB in pregnant women, the safety data for RAL and TDF are available [reviewed in van der Galien et al. (47)]. Why wait? If we know that there are no cures for HTLV-1 associated diseases, up to 24% of HTLV-1 transmission is due to MTCT, and it is those children that are most likely to develop IDH, ATLL, and/or HAM/TSP, is it not time we try and prevent this from happening by introducing RAL and TDF as a standard of care for HTLV-1 pregnant women, and in due course, potentially the later generation INSTIs like CAB?

In December last year, the FDA approved the use of long-acting injectable CAB for HIV PrEP^1^ following two large studies which illustrated that bimonthly CAB injections reduced the risk of HIV transmission more than daily PrEP pills. Given that >70% of HTLV-1 transmission occurs *via* condomless intravaginal sex, and the knowledge that INSTIs and TDF efficiently block HTLV-1 transmission in tissue culture, it is time to consider PrEP to prevent HTLV-1 transmission, and possibly prioritise CAB-based PrEP in HTLV-1 endemic settings.

Finally, whilst we urgently need to know if we can effectively block HTLV-1 sexual transmission and MTCT by using INSTIs with or without TDF, we should not forget the people who inject drugs, and patients who depend on organ donor transplantations – they too could profit from PrEP.

## Data Availability Statement

The original contributions presented in the study are included in the article/Supplementary Material, further inquiries can be directed to the corresponding author.

## Author Contributions

MB performed the *in vitro* strand transfer assays. BS did the HTLV-1 transmission experiments. GM designed and led the research. GM, BS, and MB wrote the manuscript. All authors contributed to the article and approved the submitted version.

## Conflict of Interest

The authors declare that the research was conducted in the absence of any commercial or financial relationships that could be construed as a potential conflict of interest.

## Publisher’s Note

All claims expressed in this article are solely those of the authors and do not necessarily represent those of their affiliated organizations, or those of the publisher, the editors and the reviewers. Any product that may be evaluated in this article, or claim that may be made by its manufacturer, is not guaranteed or endorsed by the publisher.

## Footnotes

1 https://www.fda.gov/news-events/press-announcements/fda-approves-first-injectable-treatment-hiv-pre-exposure-prevention, 20 December 2021.
