# Supplementary material for: Cabotegravir, the Long-Acting Integrase Strand Transfer Inhibitor, Potently Inhibits Human T-Cell Lymphotropic Virus Type 1 Transmission in vitro
Source: Front Med (Lausanne). 2022 Apr 25;9:889621. doi: 10.3389/fmed.2022.889621 (PMC9082600; doi:10.3389/fmed.2022.889621)
Supplement: Supplementary file 1 [file Table_1.DOCX]

**Supplementary Table 1.** Primer sequences.

| **Primer name** | **Sequence (5'-3')** |
| --- | --- |
| Alu-Fd | CCTCCCAAAGTGCTGGGATTACA |
| Gag-Fwd | AGCCCCCAGTTTATGCAGACC |
| Gag-Rev | GAGGGAGGAGCAAAGGTACTG |
| Gag Taqman probe | {FAM}-CTGCCAAAGACCTCCAAGACCTCC-{BHQ} |
| Albumin-Fwd | TGCATGAGAAAACGCCAGTAA |
| Albumin-Rev | ATGGTCGCCTGTTCACCAA |
| Albumin Taqman probe | {FAM}-TGACAGAGTCACCAAATGCTGCACAGAA-{BHQ} |
| Tax-Fwd | CGGATACCCAGTCTACGTGT |
| Tax-Rev | CAGTAGGGCGTGACGATGTA |
| Tax Taqman probe | {FAM}-CTGTGTACAAGGCGACTGCC-{BHQ} |
| H1U5_S20UP | AGAGAAATTTAGTACACA |
| H1U5_S20B | ACTGTGTACTAAATTTCTCT |
